# The role of circular RNA circ_0008285 in gestational diabetes mellitus by regulating the biological functions of trophoblasts

**DOI:** 10.1186/s40659-021-00337-3

**Published:** 2021-04-20

**Authors:** Haitian Chen, Shaofeng Zhang, Yanxin Wu, Zhuyu Li, Dongyu Wang, Shiqin Cai, Zilian Wang

**Affiliations:** Department of Obstetrics and Gynecology, The First Affiliated Hospital of Sun Yat-Sen University, Guangzhou, 510080 China

**Keywords:** Circular RNA, Gestational diabetes mellitus, circ_0008285, Functional analysis

## Abstract

**Background:**

Circular RNAs (circRNAs) has emerged as vital regulator involved in various diseases. In this study, we identified and investigated the potential circRNAs involved in gestational diabetes mellitus (GDM).

**Methods:**

High-throughput sequencing was used to collect the plasma circRNAs expression profiles of GDM patients. Quantitative reverse-transcriptase polymerase chain reaction (qRT-PCR) was used to measure the expressions of circ_0008285 and circ_0001173 in the plasma specimens. The Pearson’s correlation test was employed to assess the correlation between 2 circRNAs expression and the clinicopathologic data. Two circRNAs expression was verified in high glucose (HG)-induced HTR-8/SVneo cells. MTS, transwell assay was used to evaluate the effects of circ_0008285 expression on HG-induced HTR-8/SVneo cells. The network of circ_0008285 was constructed using cytocape.

**Results:**

In GDM patients, the expression of circ_0008285 was significantly upregulated, while that of circ_0001173 was decreased. Circ_0008285 was significantly correlated with the total cholesterol and LDL-C levels. Circ_0001173 was significantly correlated with glycated hemoglobin. HG promoted the proliferation, invasion, and migration in HTR-8/SVneo cells, while the knockdown of circ_0008285 exerted reverse effects. In addition, network construction exhibited that circ_0008285 had 45 miRNA binding sites, which correlated with 444 mRNA.

**Conclusions:**

circ_0008285 plays an important role and provides a clue for the usage of therapeutic targets in the development of GDM.

## Introduction

Gestational diabetes mellitus (GDM) is the first diagnosis of diabetes in pregnancy that is characterized by glucose intolerance and insulin resistance [1]. With the increase in the obesity rate and maternal age, the incidence of GDM is also increasing. Recent studies have reported [2, 3] that GDM can cause severe maternal and neonatal complications, such as increased risk of pre-eclampsia, infection, giant infants, miscarriage and premature delivery, fetal malformations, stillbirths, and cesarean section. Moreover, unfavorable maternal environment may increase the risk of fetal metabolic diseases, including type 2 diabetes, cardiovascular diseases, and obesity [4]. Presently, the diagnosis of GDM mainly depends on the diagnostic criteria of the American Diabetes Association (ADA), which is performed from 24 to 32 weeks of pregnancy [5]. In addition, the sensitivity of plasma protein profile analysis, capillary blood glucose, and other biomarkers detection methods in the second trimester of pregnancy is low [6–8]. Therefore, an early and highly sensitive biomarker is of significance for the timely diagnosis and appropriate therapeutic intervention in order to reduce the risk of adverse pregnancy outcomes among GDM patients.

Circular RNA (circRNA) is a novel type of non-coding RNA (ncRNA) that is formed through alternative splicing of pre-messenger RNA (mRNA) [7, 9]. The 3′ and 5′ ends of circRNA are connected to form a unique and complete circular covalent closed structure, which are resistant to RNA exonuclease and possess higher biological stability than that of linear RNA [8, 10]. With the development of high-throughput sequencing technology, increasing numbers of circRNAs have been discovered and their biological characteristics and regulatory functions have been revealed [9, 11]. Specific circRNAs can be used as miRNA sponges to regulate the transcription and post-transcription of miRNA-targeted genes, which play the role in several physiological and pathological processes, including proliferation, migration, and invasion [10, 12]. Termed as “microRNA sponges,” these competitive inhibitors are transcripts that are expressed from strong promoters and contain multiple, tandem- binding sites to a microRNA of interest [13]. circRNAs have close clinical relevance to human diseases such as tumors, diabetes, and cardiovascular diseases [11, 14–16]. For example, hsa_circ_0008285 has been reported to inhibit colorectal cancer cell proliferation and metastasis through sponging with miR-382-5p, promote the cervical cancer progression through sponging with miR-211-5p, and promote breast cancer progress through sponging with miR-1275 [17–19]. In addition, the hsa_circ_0001173 expression was increased in thymoma and was found to be positively correlated with immune imbalance [20]. Although some reports have indicated that circRNA may be involved in GDM [12, 16], and it is unclear whether these circRNAs are potential biomarkers. Current evidence has shown that circRNA is closely related to the occurrence and progression of GDM, preeclampsia, and other pregnancy-related diseases [14, 21, 22]. Yan et al. showed that circRNAs are abnormally expressed in the placental tissues of GDM patients, and 255 circRNAs are significantly downregulated and 227 circRNAs are significantly upregulated [15, 23]. We thus speculated that a similar circRNA phenomenon was also obvious in the GDM plasma and the peripheral blood circRNA of GDM patients may be a potential biomarker for GDM diagnosis and disease detection.

In this study, we explored the expression profiles of circRNA from the plasma of GDM women, predicted the potential functions of circRNAs in the development of GDM, and clarified the potential roles and mechanisms of circ_0008285 in GDM. Considering the post-transcriptional regulation of circ_0008285, we proposed a hypothesis that circ_0008285 may play a miRNA sponge role in the proliferation, migration, and invasion of the trophoblast cell line HTR-8/SVneo. We believe that our work may provide clues for potential early biomarkers and a novel insight to the pathological mechanism of GDM.

## Materials and methods

### Clinical specimens

The plasmas in this study were selected from 8 maternal volunteers (4 GDM cases and 4 normal controls) for sequencing and screening differentially expressed circRNAs; 120 women volunteers (57 GDM cases and 63 normal controls) were used to study the correlation between the clinicopathological data and specific circRNA expressions at the Department of Obstetrics and Gynecology, the First Affiliated Hospital of Sun Yat-Sen University, Guangzhou, China, between March and December 2019. Exclusion criteria included the delivery age < 18 years, multiple births, chronic liver and kidney diseases, diabetes, thyroid, and other endocrine diseases. GDM was diagnosed by a 75 g oral glucose tolerance test at 24–28 weeks. The clinical characteristics of the enrolled pregnant women with and without GDM are listed in Tables 1 and 2. The plasma samples of pregnant women were collected from women on an empty stomach in the early morning. After collection, the plasma samples were stored at − 80 °C. This study was approved and supervised by the Ethics Committee of First Affiliated Hospital of the Sun Yat-Sen University and signed informed consent was obtained from all volunteers.

**Table 1:** Clinical data for the GDM patients and normal controls

Characteristics	GDM (n = 4)	HC (n = 4)	p-value
Gestational age (weeks)	26.96 ± 1.32	25.25 ± 0.43	0.07
Gestational weeks of delivery	38.32 ± 1.71	38.89 ± 0.71	0.6
Pre-pregnancy BMI (kg/m^2^)	21.10 ± 0.99	23.97 ± 1.60	0.03
Fasting glucose (mmol/L)	4.23 ± 0.58	4.30 ± 0.21	0.84
1 h plasma glucose (mmol/L)	10.55 ± 0.32	7.88 ± 1.05	< 0.01
2 h plasma glucose (mmol/L)	9.33 ± 0.91	7.03 ± 0.96	0.02
GHBA1c (%)	4.90 ± 0.2	5.00 ± 0.07	0.45
Neonatal birth weight(g)	2.94 ± 0.51	3.45 ± 0.22	0.16
Hemoglobin (g/L)	108.00 ± 8.23	116.50 ± 7.5	0.23
ALT(U/L)	25.50 ± 18.68	19.50 ± 4.5	0.61
AST(U/L)	31.00 ± 12.19	19.25 ± 1.92	0.15
Triglycerides (mmol/L)	2.95 ± 1.43	1.58 ± 0.03	0.15
Total cholesterol (mmol/L)	7.08 ± 0.43	4.80 ± 0.52	< 0.01
HDL-C (mmol/L)	2.02 ± 0.14	1.71 ± 0.15	0.03
LDL-C (mmol/L)	3.88 ± 0.48	3.13 ± 0.17	0.04
TBA (μmol/L)	4.15 ± 1.13	2.58 ± 0.70	0.08

**Table 2:** Clinical data for the GDM patients and normal controls in the validation cohort

Characteristics	GDM(n = 57)	HC(n = 63)	p-value
Maternal age (year)	33.54 ± 4.62	31.90 ± 3.85	0.04
Gestational weeks of delivery	38.30 ± 8.14	38.84 ± 2.94	0.77
Pre-pregnancy BMI (kg/m^2^)	26.03 ± 1.98	26.18 ± 1.63	0.11
Fasting glucose (mmol/L)	4.45 ± 0.54	3.99 ± 0.34	< 0.001
1 h plasma glucose (mmol/L)	9.67 ± 1.27	7.40 ± 1.19	< 0.001
2 h plasma glucose (mmol/L)	8.62 ± 1.11	6.04 ± 1.17	< 0.001
GHBA1c (%)	5.40 ± 0.47	5.25 ± 0.47	0.08
Neonatal birth weight (Kg)	2.96 ± 0.58	3.13 ± 0.50	0.08
Hemoglobin (g/L)	118.10 ± 13.88	117.40 ± 12.73	0.79
ALT(U/L)	14.40 ± 6.32	15.22 ± 6.56	0.49
AST(U/L)	21.09 ± 4.69	20.43 ± 4.39	0.43
Triglycerides (mmol/L)	3.65 ± 1.44	3.69 ± 2.39	0.92
Total cholesterol (mmol/L)	6.39 ± 1.17	6.44 ± 1.02	0.81
HDL-C (mmol/L)	1.81 ± 0.32	1.80 ± 0.36	0.94
LDL-C (mmol/L)	3.65 ± 0.78	368.00 ± 0.66	0.85
TBA (umol/L)	3.44 ± 3.69	3.19 ± 1.80	0.63
Abortion history			
Yes	29 (50.88%)	15 (23.81%)	0.02
No	28 (29.12%)	48 (76.19%)	
Gestational hypertension (yes/total)			
Yes	7 (12.28%)	1 (1.59%)	0.02
No	50 (87.72%)	62 (98.41%)	
Mode of delivery			
Eutocous	30 (52.63%)	46 (73.02%)	0.02
Anesthesia operation	27 (47.37%)	17 (26.98%)	
Fetal distress			
Yes	18 (31.58%)	14 (22.22%)	0.25
No	39 (68.42%)	49 (77.78%)	
Shoulder dystocia			
Yes	1 (1.75%)	0 (0.00%)	0.29
No	56 (98.25%)	63 (100.00%)	
Preterm birth			
Yes	4 (7.55%)	7 (11.11%)	0.43
No	53 (92.45%)	56 (88.89%)	
Postpartum bleeding			
Yes	5 (8.77%)	5 (7.94%)	0.87
No	52 (91.23%)	58 (92.06%)	

### Cell culture and treatment with high glucose

The HTR-8/SVneo cells used in this study were purchased from the Committee of Type Culture Collection of Chinese Academy of Sciences. The HTR-8/SVneo cells were cultured in PRMI-1640 and supplemented with 10% fetal bovine serum (FBS) and 1% penicillin/streptomycin. In the experiment of treating HTR-8/SVneo cells with high glucose (HG), D-glucose was dissolved in the supplemented medium up to a final glucose concentration of 30.0 mmol/L [24], and then the HG medium was prepared and the HTR-8/SVneo cells were cultured in the normal medium (glucose concentration 5.0 mmol/L), which was used as a control comparison. All HTR-8/SVneo cells were incubated at 37 °C in a humidified atmosphere containing of 5% CO_2_ under standard conditions.

### Cell transfection

The HTR-8/SVneo cells (2 × 10^5^ cells/mL) were seeded into 6-well plates and incubated for 24 h until the cells reached 60–70% confluence. The cells were transfected with small-interfering RNAs (siRNAs) targeting circ_0008285 with the lipofectamine 3000 reagent according to the manufacturer’s instruction. After incubation for 4–6 h, the HTR-8/SVneo cells were further recovered with fresh medium and obtained after a period of transfection for subsequent experiments. The knockdown efficiency of siRNAs was determined by qRT-PCR. The siRNA sequences are shown in Table 3.

**Table 3:** The siRNAs sequence

Name	Sense (5′-3′)	Antisense (5′-3′)
SiRNA-1	ACGGGAAAGGUUGAAAGGAUU	AAUCCUUUCAACCUUUCCCGU
SiRNA-2	GUUAACGGGAAAGGUUGAA	UUCAACCUUUCCCGUUAAC
SiRNA-3	AACGGGAAAGGUUGAAAGG	CCUUUCAACCUUUCCCGUU
NC	AAAGCGGUUUAUGGAAAAGGG	CCCUUUUCCAUAAACCGCUUU

### Cell viability assay

Cell viability was performed using the CellTiter96 AQ Non-Radioactive Cell Proliferation Kit (Promega, Germany). The cells were trypsinized and seeded into 96-well cell culture plates at the density of 3 × 10^3^ cells/well. After the cells were incubated for 24, 48, and 72 h, 20 μL of the Solution Reagent MTS was added to the medium as per the manufacturer’s instructions (incubated at 37℃ for 0.5 h under humidified atmosphere with 5% CO_2_). Thereafter, the absorbance of the samples was measured at 490 nm using a microplate reader (Thermo Fisher, Waltham, MA, USA). All experiments were repeated more than thrice.

### Cell migration and invasion assay

For the transwell migration assay, the cells were trypsinized and adjusted to a density of 1 × 10^5^ cells/mL. The cells were added to the upper chambers of transwell (Corning, Cambridge, MA, USA) with an 8-μm pore size in a 24-well transwell insertion system to which serum-free medium was added. Meanwhile, to the lower chamber, a medium supplemented with 10% FBS was added as a chemotactic attractant. For cell invasion assay, the transwell membrane was pre-coated with 50 μL of 100% Matrigel (Corning) and then dried at 37 °C. The cells were then seeded in the upper chambers with serum-free medium. Complete medium containing 10% FBS in the lower chamber served as a chemoattractant. After 24 h of incubation at 37 ℃ with 5% CO_2_, the cells in the upper chamber were removed with a cotton swab. The migrating and invading cells through the transwell membrane were fixed in cold ethanol for 20 min and then stained with crystal violet (0.1%) for 5 min, followed by counting in at least 5 random non-overlapping regions under a light microscope (Leica, Buffalo Grove, IL, USA).

### Quantitative reverse transcriptase polymerase chain reaction (qRT-PCR)

Total RNA was isolated from the serum plasma specimen and HTR-8/SVneo cells with the TRIzol LS Reagent (Invitrogen, Carlsbad, CA, USA) according to the manufacturer’s instruction. Total RNA was separated from the supernatant as per the instruction of Cell Culture Media Exosome Purification and RNA Isolation Midi Kit (AmyJet Scientific, Wuhan, China). The purity of the extracted RNA was measured by an ultraviolet spectrophotometer (Thermo Fisher). Then, RNA was reverse-transcribed into cDNA with random primers and the SuperScript III reverse transcriptase according to the manufacturer's protocol (Qiagen, Germantown, MD, USA). PCR analysis was performed using the LightCycler 480 SYBR Green I Master Kit (Roche, Pleasanton, CA, USA) under the following conditions, 95 °C for 5 min, 45 cycles of 94 °C for 10 s, and 72 °C for 30 s. Glyceraldehyde 3-phosphate dehydrogenase (GAPDH) was used as the internal control. Primer sequences are listed in Table 4. The expression levels of circRNAs in serum plasma specimen were calculated using delta CT, and the relative expression of circRNAs in HTR-8/SVneo cells and cells supernatant were calculated using the 2^−ΔΔCt^ method after normalization with the reference control [25].

**Table 4:** The primer sequence of circRNAs

Primer name	Sequence (5′-3′)
GAPDH-divergent-F	TCCTCACAGTTGCCATGTAGACCC
GAPDH-divergent-R	TGCGGGCTCAATTTATAGAAACCGGG
GAPDH-convergent-F	GAGTCAACGGATTTGGTCGT
GAPDH-convergent-R	GACAAGCTTCCCGTTCTCAG
circ_0008285-divergent-F	TCATAGCCTTTCCACCGA
circ_0008285-divergent-R	ACAGTGGCACCCGAAGTG
circ_0008285-convergent-F	ACCTCTCCTAAGGCACTCGT
circ_0008285-convergent-F	GGTCCAGTTTCTCAGGGCTC
circ_0001173-divergent-F	GCTGGCAATTCAAACACACA
circ_0001173-divergent-R	CTACGGGAGGAGAACAAGCA
circ_0001173-convergent-F	TGTGTGTTTGAATTGCCAGC
circ_0001173-convergent-F	TGCTTGTTCTCCTCCCGTAG

### RNase R treatment

RNase R was purchased from Geneseed Biotech Co., Ltd. (Guangzhou, China). RNase R treatment was performed as described in a previous study [26]. In brief, total RNA (2 μg) was incubated with RNase R (3 U/μg) for 10 min at 37 °C. Then, RNA was used for qRT-PCR to evaluate the stability of circ_0008285 and circ_0001173.

### Sanger sequencing

Divergent and convergent primers were used to verify the backsplice junction of circRNA (Table 4). PCR was performed according to the manufacturer's protocol for Premix Taq (TaKaRa, Madison, WI, USA). The cDNA and gDNA PCR products were visualized by gel electrophoresis on 4% ethidium bromide-stained agarose gels. The bands were examined by UV irradiation (UVP Inc., San Gabriel, CA, USA). The PCR products of divergent primers were subjected to Sanger sequencing by Sangon (Shanghai, China).

### Bioinformatics analysis

Total RNA was isolated from the plasma by using the miRNeasy Serum/Plasma Kit (QIAGEN, Duesseldorf, Germany). We removed ribosomal RNA with the VAHTS Total RNA-seq (H/M/R) Library Prep Kit from Illumina (Vazyme Bio-tech Co.; Ltd., Nanjing, China). Next, RNA was incubated with RNase R at 37℃ and then RNA-Seq libraries were constructed using the KAPA Stranded RNA-Seq Library Prep Kit (Roche, Basel, Switzerland), which was used for deep sequencing on the platform of Illumina HiSeq 2500 (Illumina). We used Tophat2 to map the reading segments to the reference genome. TopHat-fusion were used to assemble the mapped reads from the circRNA de novo and recognized the back-splicing reads in the unmapped reads. Differentially expressed genes (DEGs) were defined as those expressed either log2 (fold change) ≥ 0.67 or log2 (fold change) ≤  − 0.67 in the GDM group when compared to that in the healthy pregnant individuals with a statistical significance (p < 0.01). Volcano map and heat map analysis were used to distinguish information between the 2 groups.

### Network of hsa_circ_0008285

To obtain the interaction network of hsa_circ_0008285 with miRNA and target mRNA, we used miRanda (http://www.microrna.org) to predict the relationships between the circRNA and miRNA [27] and Cytoscape tool (http://cytoscape.org) to construct a network map of hsa_circ_0008285-miRNA-mRNA.

### Kyoto Encyclopedia of Genes and Genomes (KEGG) biological pathway analysis

Predicted mRNAs in the network of hsa_circ_0008285 were further analyzed based on the function by DAVID, bioinformatics resources V6.8 tool with its KEGG biological pathway analyses [28]. Enrichment gene count ≥ 2 and hypergeometric test significance threshold P < 0.05 were considered to be a significant correlation.

### Statistical analysis

Statistical analysis of the data was performed with the SPSS version 21.0 (SPSS Inc, Chicago, IL, USA) and GraphPad Prism 5.0 (GraphPad Software Inc, San Diego, CA, USA). Data was described as mean ± standard deviation. Student's *t*-test or χ2 test was used to assess the differences between the experimental groups. The correlation between 2 variables was analyzed by using the Pearson correlation analysis. Differences with p < 0.05 were considered to be statistically significant.

## Results

### Overview of plasma circRNAs in GDM patients

**Fig. 1:**
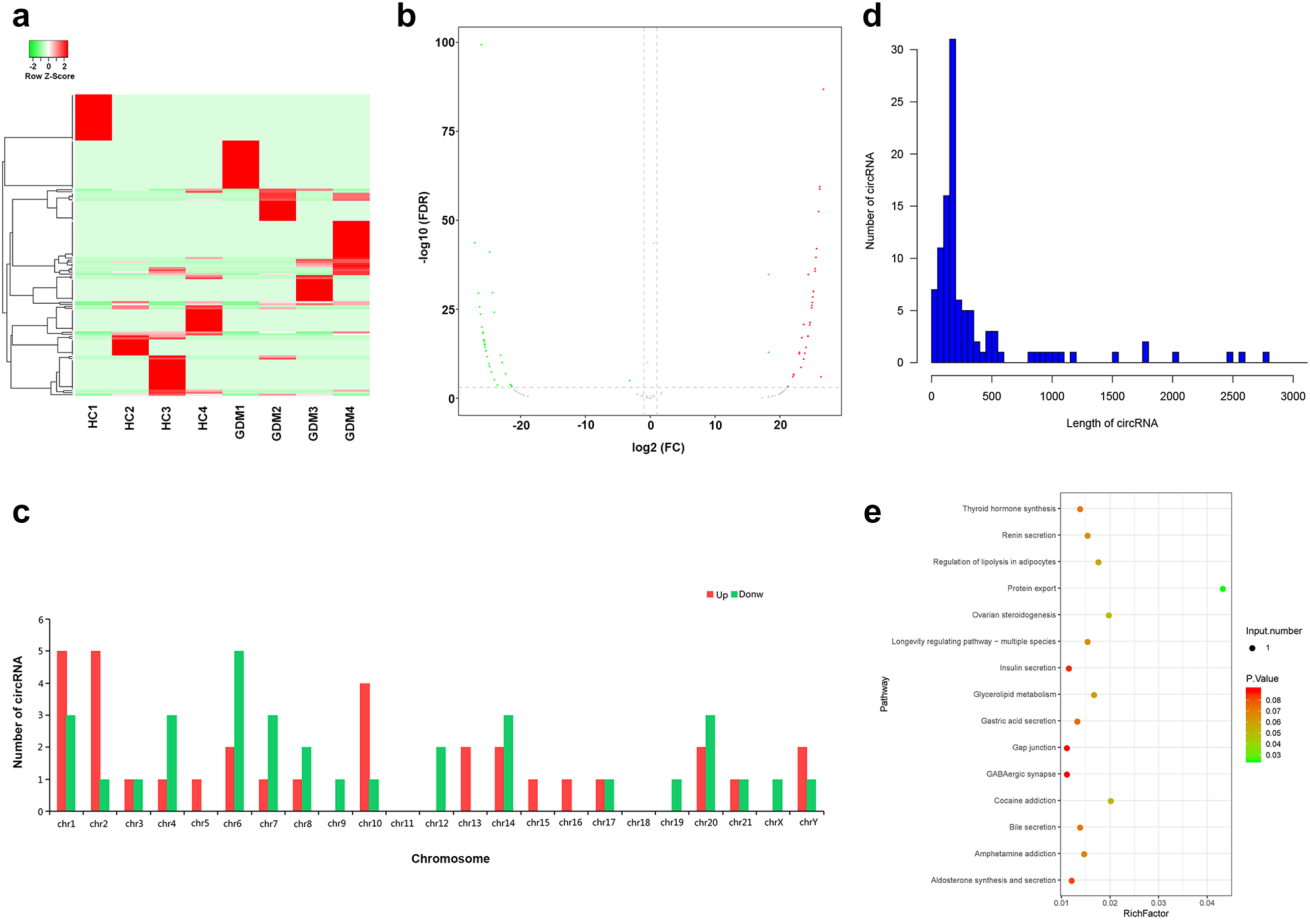
Differentially expressed circular RNAs (circRNAs) in the plasma. **a** Hierarchical cluster analysis of differential circRNAs. The color scale of the strips runs from green (low relative expression) to red (high relative expression). **b** Volcano map visualizing the differentially expressed circRNAs in the plasma. The red and green plots represent the significantly differentially expressed upregulated and downregulated circRNAs, respectively (log2 [fold change] ≥ 0.67, P < 0.05). **c** The chromosome distributions of differentially expressed circRNAs. The red and green bands indicate the distribution of upregulated and downregulated circRNAs in the chromosomes, respectively. **d** Length enrichment distribution map of differentially expressed circRNAs. The length of the significantly differentially expressed circRNA is mainly within 500 bp. **e** The KEGG pathway enrichment analysis of parent gene with significantly differentially expressed circRNAs

In order to determine which circRNA can be used as potential early biomarkers and to obtain a novel insight into the pathological mechanisms of GDM, a preliminary analysis of all the sequencing results were performed. Hierarchical cluster analysis and Volcano map revealed the circRNA expression levels in the plasma from the GDM and normal controls (Fig. 1a, b). In total, 147 circRNAs were screened as differentially expressed circRNAs. Among these, 76 circRNAs were upregulated and 71 circRNAs were downregulated between the 2 groups (Fig. 1a, b). These data indicated that circRNAs in GDM were different from those in the HC group. In addition, Fig. 1c revealed the chromosome distributions of these differential circRNAs. The upregulated circRNAs were mainly distributed on chromosomes 1, 2, and 10, while the downregulated circRNAs were mainly distributed on chromosomes 6. Furthermore, Fig. 1d depicted that the length of most differential circRNAs were mainly within 500 bp. In order to study the biological functions of these significantly differentially expressed cricRNAs, the KEGG pathway enrichment analysis was preformed, which indicated that the pathways related to protein transport were significantly enriched (Fig. 1e).

### Validation of circRNA expression

**Fig. 2:**
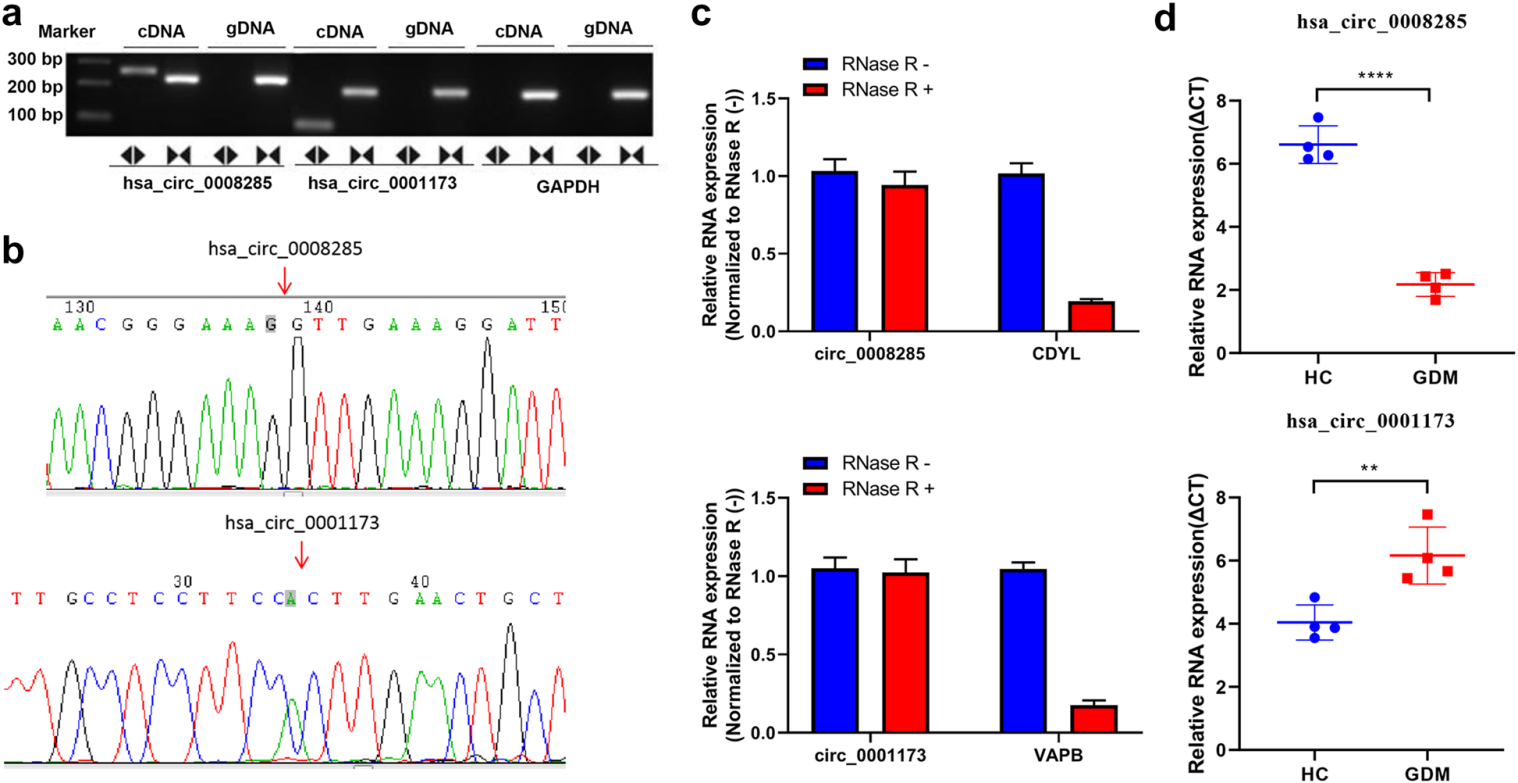
Validation of circ_0008285 and circ_0001173 in sequencing samples. **a** The circularity of circ_0008285 and circ_0001173 verified in the HTR-8/SVneo cells by RT-PCR. circ_0008285 and circ_0001173 were amplified by divergent primers in cDNA and not in gDNA. **b** Sanger sequence analysis was used to confirm the splicing site. **c** The stability of circ_0008285 and circ_0001173 were evaluated via the RNase R treatment experiment. **d** The expression levels of the circ_0008285 and circ_0001173 were validated by qRT-PCR. GDM, gestational diabetes mellitus; HC, healthy pregnant controls. **P < 0.01, ****P < 0.0001

In the differential circRNAs, we further analyzed the DEGs with expressed log2 (fold change) > 2 and p < 0.01, and we selected 2 circRNAs, which have been recorded in circbase (http://www.circbase.org/) for further study. First, we validated the circularity of circ_0008285 and circ_0001173 in the HTR-8/SVneo cells, and the results are shown in Fig. 2a and b. We also tested the stability of circ_0008285 and circ_0001173 via RNase R treatment experiment, and the results revealed that the relative circ_0008285 and circ_0001173 expressions showed no obvious change after RNase R treatment, whereas their response parent gene CDYL and VAPB expressions decreased significantly (Fig. 2c). Then, the relative expression levels of the circ_0008285 and circ_0001173 were measured by RT-qPCR in the sequencing samples, and their expression patterns were similar to those observed in the sequencing results (Fig. 2d). These results indicated that the expression of circ_0008285 was significantly upregulated, while that of circ_0001173 was significantly reduced in GDM, which confirmed the head-to-tail splicing in circ_0008285 and circ_0001173.

### Correlation analyses of differentially expressed circRNAs and clinical indicators

**Fig. 3:**
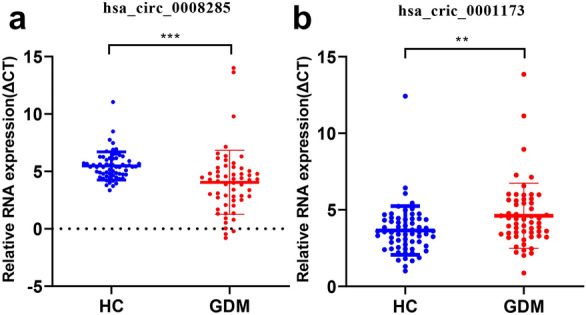
Validation of circ_0008285 and circ_0001173 in 120 women volunteers. The expression levels of the circ_0008285 and circ_0001173 were validated by qRT-PCR. GDM, gestational diabetes mellitus; HC, healthy pregnant controls. **P < 0.01, ***P < 0.001

To further study the expressions of circ_0008285 and circ_0001173 in the plasma of GDM patients, qRT-PCR was performed. Among the included patients, the blood glucose levels (Fasting glucose, 1 h and 2 h plasma glucose) of the GDM group were higher than those of the HC group (P < 0.05). Abortion history, gestational hypertension, and the mode of delivery were significantly different between the GDM group and the HC group (Table 2). When compared with the HC group, the expression levels of circ_0008285 were significantly increased, while those of circ_0001173 were significantly decreased in the GDM group (Fig. 3). These results are consistent with those of Fig. 2d. Next, we analyzed the correlation between the levels of candidate circRNAs and clinical indicators in GDM maternal. The level of circ_0008285 was significantly correlated with the total cholesterol and LDL-C values, and the correlation coefficients were 0.370 and 0.368, respectively (P < 0.05). circ_0001173 was expressed during the second trimester and its level in the plasma was correlated with that of the glycated hemoglobin (GHBA1c) (Table 5). These results indicated that these 2 circRNAs may play an important role in GDM.

**Table 5:** Correlation among the clinicopathologic status and the expressions of has_circ_0008285 and has_cric_0001173 in GDM patients

Characteristics	Numbers	has_cric_0008285		has_circ_0001173	
		R value	p value	R value	p value
Maternal age (year)	57	− 0.02	0.87	− 0.03	0.85
Gestational weeks of delivery	57	− 0.08	0.56	− 0.02	0.87
Pre-pregnancy BMI (kg/m^2^)	57	− 0.23	0.09	− 0.05	0.70
Fasting glucose (mmol/L)	57	− 0.01	0.92	0.12	0.34
1 h plasma glucose (mmol/L)	57	− 0.08	0.55	0.11	0.43
2 h plasma glucose (mmol/L)	57	0.02	0.89	− 0.18	0.19
GHBA1c (%)	57	0.22	0.10	0.32*	0.02
Neonatal birth weight (Kg)	57	− 0.17	0.20	− 0.85	0.53
Hemoglobin (g/L)	57	0.20	0.14	0.18	0.19
ALT (U/L)	57	0.01	0.97	0.11	0.43
AST (U/L)	57	− 0.03	0.84	− 0.06	0.64
Triglycerides (mmol/L)	57	0.16	0.24	0.00	1.00
Total cholesterol (mmol/L)	57	0.37**	0.01	0.02	0.91
HDL-C (mmol/L)	57	0.13	0.35	− 0.20	0.14
LDL-C (mmol/L)	57	0.368**	0.05	− 0.05	0.74
TBA (μmol/L)	57	− 0.09	0.50	0.02	0.91
Abortion history					
Yes	29	− 0.12	0.38	0.01	0.93
No	28				
Gestational hypertension					
Yes	7	− 0.16	0.23	− 0.13	0.35
No	50				
Mode of delivery					
Eutocous	30	− 0.21	0.13	− 0.07	0.59
Anesthesia operation	27				
Fetal distress					
Yes	18	0.07	0.63	0.11	0.42
No	39				
Shoulder dystocia					
Yes	1	0.10	0.46	− 0.26	0.05
No	56				
Preterm birth					
Yes	4	− 0.08	0.56	− 0.02	0.86
No	53				
Postpartum bleeding					
Yes	5	0.10	0.46	− 0.26	0.05
No	52				

### Circ_0008285 is upregulated in high glucose-treated HTR-8/SVneo cells

**Fig. 4:**
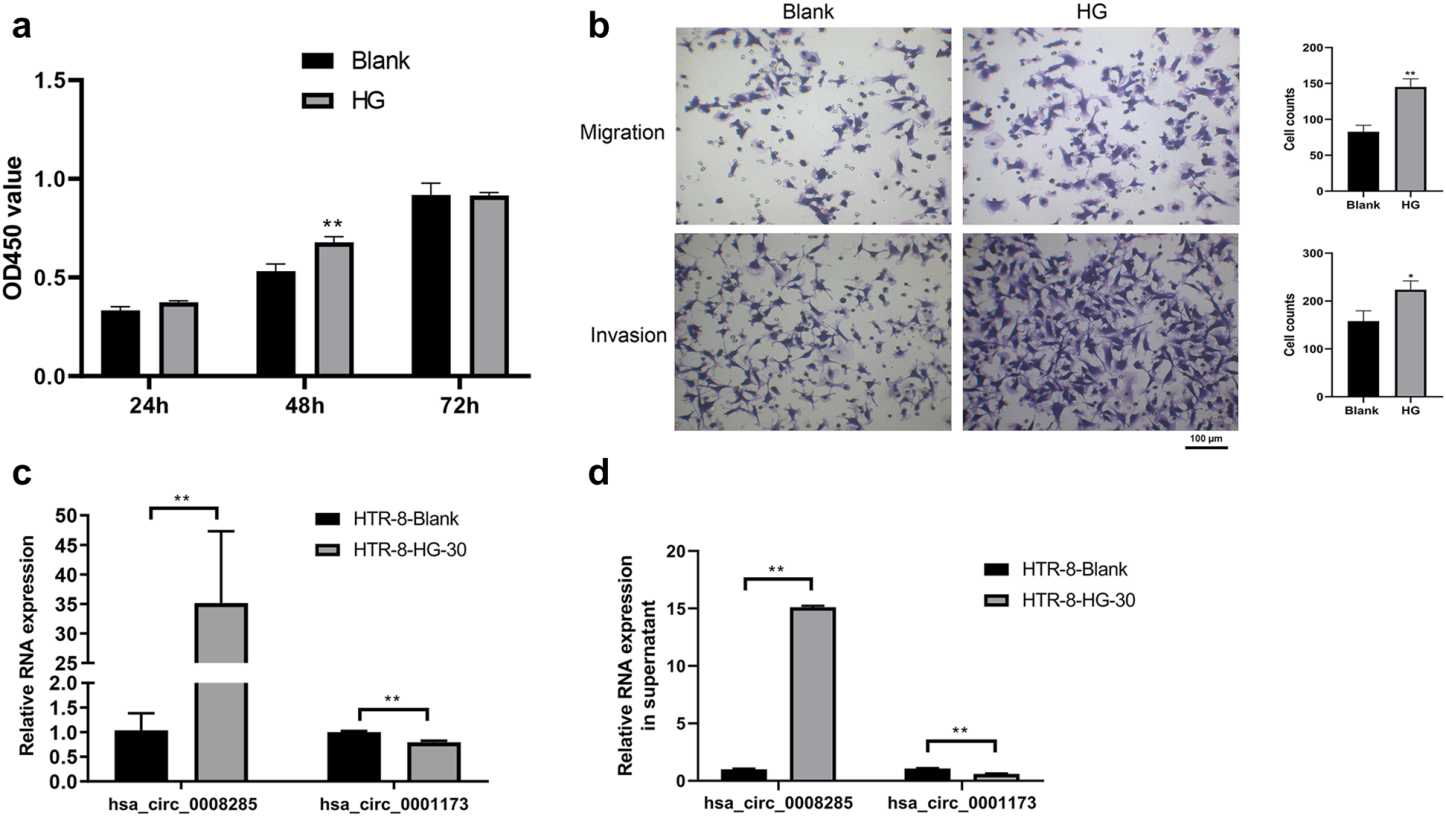
High glucose treatment enhances the cytological functions and affects the expression levels of circ_0008285 and circ_0001173. **a** High glucose promotes the HTR-8/SVneo cells proliferation. **b** The migration and invasion of HTR-8/SVneo cells were promoted by high glucose treatment. **c** High glucose significantly upregulated the level of circ_0008285 and decreased the level of circ_0001173 in the HTR-8/SVneo cells. **d** The expression of circ_0008285 was increased and the expression of circ_0001173 was decreased in the supernatant after HTR-8/SVneo cells were treated with HG. HG, high glucose. Mean ± SEM (n = 3), *P < 0.05, **P < 0.01

In order to study the role of circRNAs in GDM, the HTR-8/SVneo cells were treated with a HG medium for 24 h under in vitro conditions. The results (Fig. 4a, b) revealed that the proliferation, migration, and invasion of the HTR-8/SVneo cells were promoted by HG treatment when compared with those of cells under the normal condition. Accordingly, qRT-PCR analysis was performed to determine the expression levels of circ_0008285 and circ_0001173. The results as shown in Fig. 4c, which indicate that treatment with HG significantly upregulated the level of circ_0008285 and decreased the level of circ_0001173 in the HTR-8/SVneo cells (P < 0.05). Furthermore, circ_0008285 and circ_0001173 expressions were also detected in the supernatant, and the expression trend was found to be consistent with those of the HTR-8/SVneo cells after HG treatment (Fig. 4d). circ_0008285 was selected for further research as its expression was more significant between the HG group and under the normal condition relative to circ_0001173.

### Downregulation of circ_0008285 suppresses trophoblast proliferation, migration, and invasion

**Fig. 5:**
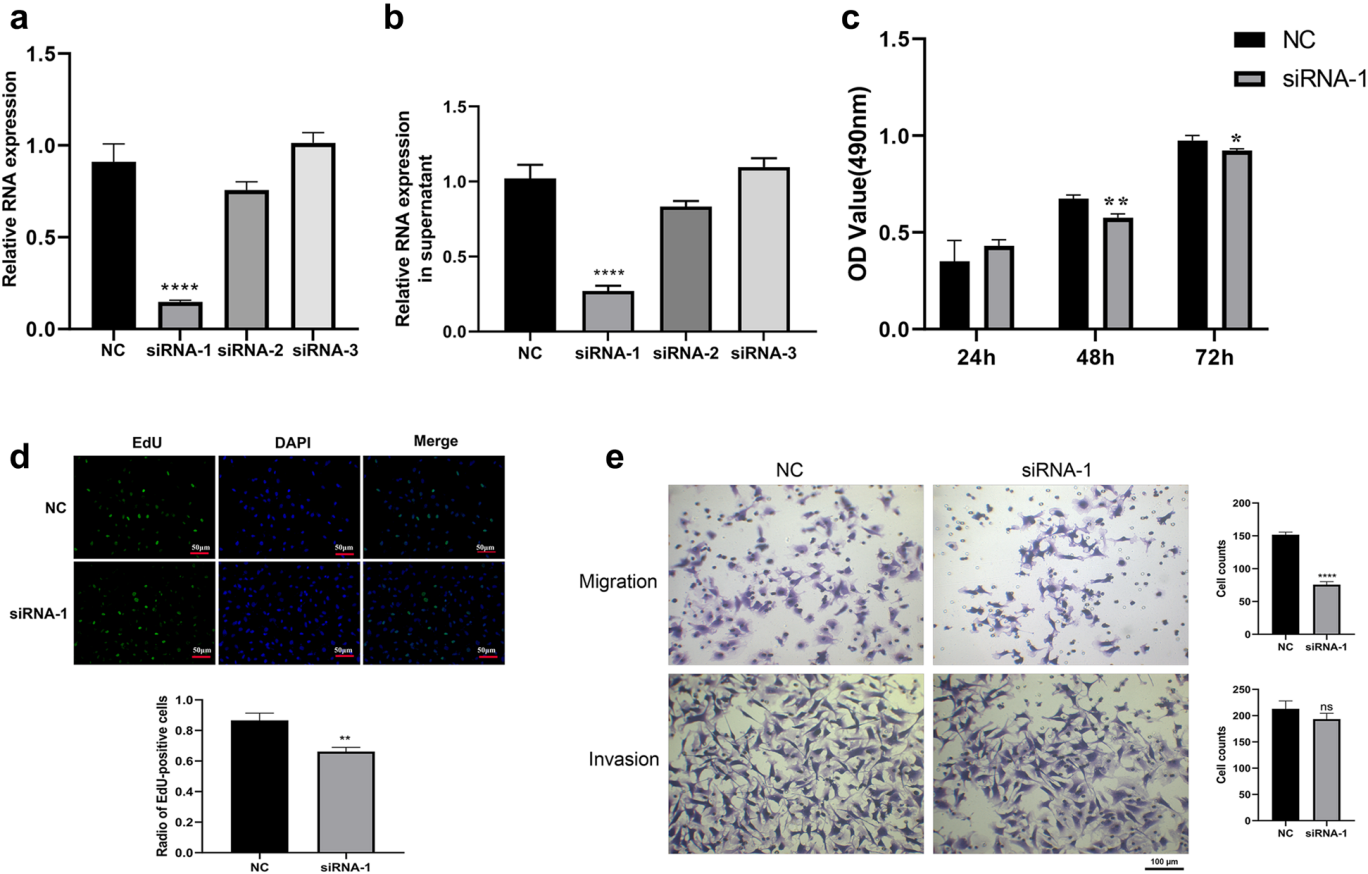
Downregulation of circ_0008285 suppresses proliferation, migration, and invasion of HTR-8/SVneo cells under high glucose condition. **a** Confirmed inhibition effect of siRNAs on the circ_0008285 expression in HTR-8/SVneo cells. **b** Confirmation of the expression of circ_0008285 in the supernatant after HTR-8/SVneo cells transfection with siRNAs. **c** Silencing of circ_0008285 inhibits the proliferation of HTR-8/SVneo cells cultivated in high glucose medium. **d** EdU staining confirms the inhibition effects of HTR-8/SVneo cell proliferation by silencing circ_0008285 under high glucose treatment. **e** Silencing of circ_0008285 inhibits the migration and invasion of HTR-8/SVneo cells in high glucose. NC, siNC. Mean ± SEM (n = 3), *P < 0.05, **P < 0.01, ****P < 0.0001

To further explore the functions of circ_0008285 in trophoblast cells, siRNA-1, siRNA-2, and siRNA-3 targeting circ_0008285 were transfected into the human trophoblast cell line HTR-8/SVneo, and siRNA-1 was shown to significantly reduce the expression of circ_0008285, whereas the expression of circ_0008285 showed no obvious expression change after transfection with siRNA-2 and siRNA-3 (Fig. 5a). These expression changes were also observed in the supernatant (Fig. 5b). Then, the cytological functions were determined in the HTR-8/SVneo cells (siRNA-1 and si-NC groups) cultivated in the HG medium. The proliferation ability of HTR-8/SVneo cells that interfered with siRNA-1 was significantly inhibited (Fig. 5c, d). The results of transwell assays (Fig. 5e) revealed that the migration and invasion capabilities of HTR-8/SVneo cells was reduced in the si-circRNA-1 group when compared to that in the si-NC control group. These results indicate that the downregulation of circ_0008285 suppressed the proliferation, migration, and invasion of the HTR-8/SVneo cells.

### Prediction of potential mechanisms of hsa_circ_0008285

**Fig. 6:**
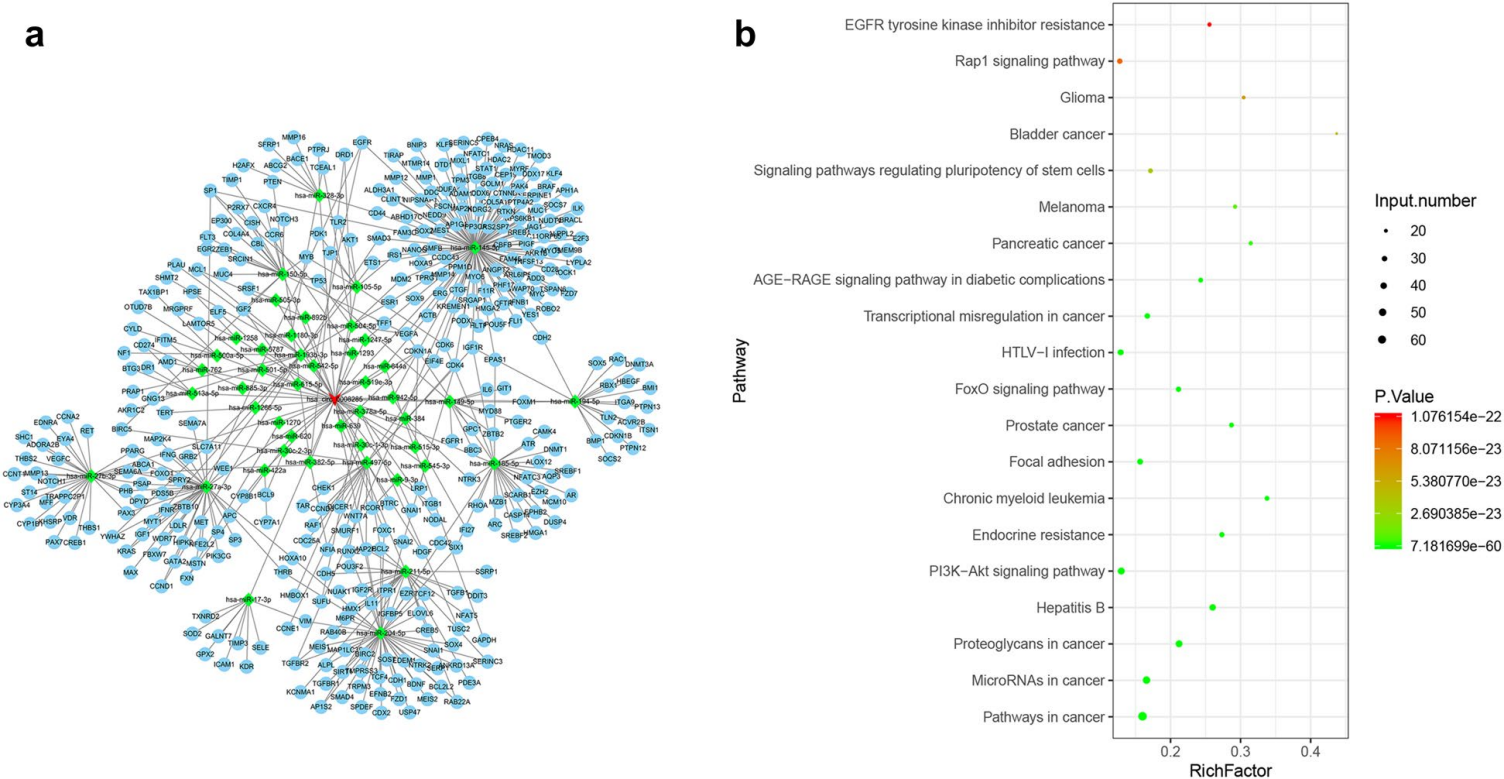
MiRanda and KEGG biological pathway analyses were conducted to preliminarily explore the possible mechanisms. **a** circ_0008285-miRNA-mRNA network predicted by miRanda. **b** A scatter plot for the top 20 of the KEGG pathway for mRNAs in the network

In order to further study the potential mechanism of hsa_circ_0008285, we used miRanda to predict the interaction among circ_0008285, miRNA, and its target mRNA (Fig. 6a). We found that 45 miRNAs and 444 mRNAs may be regulated by circ_0008285 (Fig. 6a). Furthermore, we performed pathway enrichment analysis of these mRNAs based on the KEGG database (Fig. 6b), the top of 20 KEGG enrichment pathway indicated that most of the mRNAs were mainly enriched in cancer pathways, the PI3K/AKT pathway, and miroRNAs in cancer. This result revealed the potential mechanism of hsa_circ_0008285, which warrants further validation.

## Discussion

GDM is characterized by different degrees of abnormal glucose metabolism during pregnancy, which causes a variety of serious complications including dystocia, giant infants, and neonatal hypoglycemia [29, 30]. Similar to other studies, we also found that the GDM group had higher blood glucose level and a higher incidence of abortion and gestational hypertension when compared to those in the healthy pregnant control. New diagnoses and treatment method is of great importance to avoid poor pregnancy outcomes. In our study, the aberrant expression of circRNAs in GDM patient’s plasma was recorded and significant association was observed between altered circRNAs and GDM-related pathological contexts.

CircRNA is a type of non-coding RNA molecule with a closed circular structure [9]. With the increase in high-throughput sequencing technology, the number of circRNAs reported in the recent years has dramatically increased, revealing its important regulatory functions and its potential value as a target for the diagnosis and treatment of various diseases. From the perspective of mechanism, circRNAs are considered to participate in gene regulation by acting as a sponge of miRNAs, which limits their inhibition of target genes [31]. Wang et al. [21] examined the differential expression of circRNAs in the placentas of GDM women through RNA sequencing analysis and found that the expressions of circ_0005824, circ_0003636, and circ_0000395 in the GDM group were significantly lower than those in the control group. Our study was the first to screen differential expression profiles of circRNAs in the plasma between GDM and normal pregnant women in order to explore the relationship between circRNAs and GDM development. The results revealed 147 differentially expressed circRNAs in the GDM group when compared with that in the healthy pregnant controls, indicating that the expression patterns of circRNAs in GDM were different from those in healthy pregnant women.

The biological functions and the potential pathways of the parental genes of circRNAs that were significantly differentially expressed were preliminarily predicted by the KEGG pathway analysis, and the pathway related to protein transport was found to be closely related [32]. In this study, we found that the expression of circ_0008285 was significantly increased and positively correlated with the total cholesterol and low-density lipoprotein levels, while the expression of circ_0001173 was significantly downregulated and positively correlated with the glycosylated hemoglobin level in GDM patients. Furthermore, circ_0008285 was selected for further studies because the upregulation of circ_0008285 was more obvious than the downregulation of circ_0001173. circ_0008285 may participate in the development of GDM by affecting cell metabolisms and cell cycles during pregnancy. Past studies have indicated that has_circ_0008285 can competitively bind to miR-211-5p as a miRNA sponge, release SOX4, and promote the self-renewal and proliferation of cervical cancer cells [18]. It is known that the proliferation of trophoblast cells is increased during hyperglycemia and that maternal diabetic women have higher placenta weight [33]. However, the biological functions of circ_0008285 on HTR-8/SVneo trophoblast cells in GDM has not yet been reported. We found that the proliferation, migration, and invasion abilities of HTR-8/SVneo cells treated with HG in vitro were inhibited after the circ_0008285 knockdown, and circ_0008285 harbored one or more binding sites for GDM-related miRNAs, such as hsa-miR-145-5p, hsa-miR-240-5p, and hsa-miR-211-5p. Thus, we speculated that the roles of circ_0008285 in GDM may be related to miRNA-mediated effects. The underlying mechanisms of the circRNA-miRNA interaction needs further analyses.

In order to explore the underlying mechanisms of circ_0008285 activities, we constructed a network of circRNA-miRNA-mRNA and then analyzed these mRNAs in terms of the pathways involved. The results suggested that circ_0008285 plays a role through several pathways, including the PI3K/Akt signaling pathway. It has been confirmed that the PI3K/Akt pathway activation plays an important role in promoting placental development, fetal growth, and GDM in humans and rodents [34, 35]. Moreover, the PI3K/Akt signaling pathway is important for cell proliferation, migration, invasion, and gluconeogenesis [36]. Past studies have demonstrated that the inhibition of the PI3K/Akt signaling pathway can reduce oxidative stress, affect the function of trophoblast cells, and reduce the blood glucose level in a GDM rat model [37]. Based on these abovementioned results and predictions, we speculated that circ_0008285 may play an important role in maintaining the HTR-8/SVneo trophoblast cell function and promoting the development of GDM through the PI3K/Akt signaling pathway. Further studies are warranted to gain a deeper understanding of this regulatory mechanism. Moreover, the expression of circ_0008285 also requires further validation for the placental tissues.

## Conclusions

Taken together, the results of this study showed, for the first time, that the expression of circRNAs in the plasma of GDM patients was significantly different from those in normal pregnant women, while the expression of circ_0008285 was significantly upregulated in GDM patients. In vitro experiments confirmed that the downregulation of circ_0008285 inhibited the proliferation, migration, and invasion of trophoblast HTR-8/SVneo cells. According to the analysis of circRNA-miRNA-mRNA network and KEGG analysis of mRNAs in this network, we assumed that circ_0008285 may participate in GDM regulation by activating the PI3K/Akt pathway. Although circ_0008285 may provide a clue for a new potentially sensitive serum biomarker for GDM, owing to the complexity of its regulatory mechanism, it is recommended to conduct more in-depth in vivo and in vitro studies.

## Data Availability

All data generated or analyzed during this study are included in this published article.

## Abbreviations

circRNAs: Circular RNAs
GDM: Gestational diabetes mellitus
qRT-PCR: Quantitative reverse transcriptase polymerase chain reaction
HG: High glucose
ADA: American Diabetes Association
ncRNA: Non-coding RNA
FBS: Fetal bovine serum
DEGs: Differentially expressed genes

## References

[1] Alejandro EU, Mamerto TP, Chung G, Villavieja A, Gaus NL, Morgan E, Pineda-Cortel MRB (2020). Gestational diabetes mellitus: a harbinger of the vicious cycle of diabetes. Int J Mol Sci..

[2] Damm P, Houshmand-Oeregaard A, Kelstrup L, Lauenborg J, Mathiesen ER, Clausen TD (2016). Gestational diabetes mellitus and long-term consequences for mother and offspring: a view from Denmark. Diabetologia.

[3] Retnakaran R, Luo J, Shah BR (2019). Gestational diabetes in young women predicts future risk of serious liver disease. Diabetologia.

[4] Lei Q, Niu J, Lv L, Duan D, Wen J, Lin X, Mai C, Zhou Y (2016). Clustering of metabolic risk factors and adverse pregnancy outcomes: a prospective cohort study. Diabetes Metab Res Rev.

[5] Goyal A, Gupta Y, Singla R, Kalra S, Tandon N (2020). American Diabetes Association "Standards of Medical Care-2020 for Gestational Diabetes Mellitus": a critical appraisal. Diabetes Ther.

[6] Zhao C, Wang F, Wang P, Ding H, Huang X, Shi Z (2015). Early second-trimester plasma protein profiling using multiplexed isobaric tandem mass tag (TMT) labeling predicts gestational diabetes mellitus. Acta Diabetol.

[7] García-Claver A, Ramos-Corral R, Laviña-Fañanás C, Solans-Blecua I, Puzo-Foncillas J (2020). Capillary glucose concentration during oral glucose tolerance test for the diagnosis of gestational diabetes. Int J Gynaecol Obstet.

[8] Amini M, Kazemnejad A, Zayeri F, Montazeri A, Rasekhi A, Amirian A, Kariman N (2020). Diagnostic accuracy of maternal serum multiple marker screening for early detection of gestational diabetes mellitus in the absence of a gold standard test. BMC Pregnancy Childbirth.

[9] Chen B, Huang S (2018). Circular RNA: an emerging non-coding RNA as a regulator and biomarker in cancer. Cancer Lett.

[10] Awan FM, Yang BB, Naz A, Hanif A, Ikram A, Obaid A, Malik A, Janjua HA, Ali A, Sharif S (2021). The emerging role and significance of circular RNAs in viral infections and antiviral immune responses: possible implication as theranostic agents. RNA Biol.

[11] Li Y, Ashraf U, Chen Z, Zhou D, Imran M, Ye J, Chen H, Cao S (2020). Genome-wide profiling of host-encoded circular RNAs highlights their potential role during the Japanese encephalitis virus-induced neuroinflammatory response. BMC Genomics.

[12] Mumtaz PT, Taban Q, Dar MA, Mir S, Haq ZU, Zargar SM, Shah RA, Ahmad SM (2020). Deep insights in Circular RNAs: from biogenesis to therapeutics. Biol Proced Online.

[13] Ebert MS, Neilson JR, Sharp PA (2007). MicroRNA sponges: competitive inhibitors of small RNAs in mammalian cells. Nat Methods.

[14] Yang X, Mei J, Wang H, Gu D, Ding J, Liu C (2020). The emerging roles of circular RNAs in ovarian cancer. Cancer Cell Int.

[15] Jakobi T, Siede D, Eschenbach J, Heumüller AW, Busch M, Nietsch R, Meder B, Most P, Dimmeler S, Backs J, Katus HA, Dieterich C (2020). Deep characterization of circular RNAs from human cardiovascular cell models and cardiac tissue. Cells..

[16] Filardi T, Catanzaro G, Mardente S, Zicari A, Santangelo C, Lenzi A, Morano S, Ferretti E (2020). Non-coding RNA: role in gestational diabetes pathophysiology and complications. Int J Mol Sci..

[17] Wang J, Luo J, Liu G, Li X (2020). Circular RNA hsa_circ_0008285 inhibits colorectal cancer cell proliferation and migration via the miR-382-5p/PTEN axis. Biochem Biophys Res Commun.

[18] Bai Y, Li X (2020). hsa_circ_0008285 facilitates the progression of cervical cancer by targeting miR-211-5p/SOX4 Axis. Cancer Manag Res.

[19] Liang G, Ling Y, Mehrpour M, Saw PE, Liu Z, Tan W, Tian Z, Zhong W, Lin W, Luo Q, Lin Q, Li Q, Zhou Y, Hamai A, Codogno P, Li J, Song E, Gong C (2020). Autophagy-associated circRNA circCDYL augments autophagy and promotes breast cancer progression. Mol Cancer.

[20] Wu Q, Luo X, Li H, Zhang L, Su F, Hou S, Yin J, Zhang W, Zou L (2021). Identification of differentially expressed circular RNAs associated with thymoma. Thorac Cancer..

[21] Wang H, Zhou W, She G, Yu B, Sun L (2020). Downregulation of hsa_circ_0005243 induces trophoblast cell dysfunction and inflammation via the β-catenin and NF-κB pathways. Reprod Biol Endocrinol.

[22] Wang H, She G, Zhou W, Liu K, Miao J, Yu B (2019). Expression profile of circular RNAs in placentas of women with gestational diabetes mellitus. Endocr J.

[23] Yan L, Feng J, Cheng F, Cui X, Gao L, Chen Y, Wang F, Zhong T, Li Y, Liu L (2018). Circular RNA expression profiles in placental villi from women with gestational diabetes mellitus. Biochem Biophys Res Commun.

[24] Peng HY, Li HP, Li MQ (2020). Downregulated ABHD5 aggravates insulin resistance of trophoblast cells during gestational diabetes mellitus. Reprod Sci.

[25] Adnan M, Morton G, Hadi S (2011). Analysis of rpoS and bolA gene expression under various stress-induced environments in planktonic and biofilm phase using 2(-ΔΔCT) method. Mol Cell Biochem.

[26] Niu Q, Dong Z, Liang M, Luo Y, Lin H, Lin M, Zhong X, Yao W, Weng J, Zhou X (2020). Circular RNA hsa_circ_0001829 promotes gastric cancer progression through miR-155-5p/SMAD2 axis. J Exp Clin Cancer Res.

[27] Akgül B, Göktaş C (2014). Gene reporter assay to validate microRNA targets in Drosophila S2 cells. Methods Mol Biol.

[28] Huang DW, Sherman BT, Tan Q, Kir J, Liu D, Bryant D, Guo Y, Stephens R, Baseler MW, Lane HC, Lempicki RA (2007). DAVID boinformatics resources: expanded annotation database and novel algorithms to better extract biology from large gene lists. Nucleic Acids Res.

[29] Magee TR, Ross MG, Wedekind L, Desai M, Kjos S, Belkacemi L (2014). Gestational diabetes mellitus alters apoptotic and inflammatory gene expression of trophobasts from human term placenta. J Diabetes Complications.

[30] Meghelli L, Vambergue A, Drumez E, Deruelle P (2020). Complications of pregnancy in morbidly obese patients: what is the impact of gestational diabetes mellitus?. J Gynecol Obstet Hum Reprod.

[31] Ren S, Lin P, Wang J, Yu H, Lv T, Sun L, Du G (2020). Circular RNAs: promising molecular biomarkers of human aging-related diseases via functioning as an miRNA sponge. Mol Ther Methods Clin Dev.

[32] Du J, Yuan Z, Ma Z, Song J, Xie X, Chen Y (2014). KEGG-PATH: Kyoto encyclopedia of genes and genomes-based pathway analysis using a path analysis model. Mol Biosyst.

[33] Wu Z, Mao W, Yang Z, Lei D, Huang J, Fan C, Suqing W (2021). Knockdown of CYP1B1 suppresses the behavior of the extravillous trophoblast cell line HTR-8/SVneo under hyperglycemic condition. J Matern Fetal Neonatal Med.

[34] Ye HH, Yang SH, Zhang Y (2018). MEG3 damages fetal endothelial function induced by gestational diabetes mellitus via AKT pathway. Eur Rev Med Pharmacol Sci.

[35] Li HX, Li XH, Jiang J, Shi PX, Zhang XG, Tian M (2020). Effect of miR-26b on gestational diabetes mellitus in rats via PI3K/Akt signaling pathway. Eur Rev Med Pharmacol Sci.

[36] Xing Y, Ren S, Ai L, Sun W, Zhao Z, Jiang F, Zhu Y, Piao D (2019). ZNF692 promotes colon adenocarcinoma cell growth and metastasis by activating the PI3K/AKT pathway. Int J Oncol.

[37] Zong HY, Wang EL, Han YM, Wang QJ, Wang JL, Wang Z (2019). Effect of miR-29b on rats with gestational diabetes mellitus by targeting PI3K/Akt signal. Eur Rev Med Pharmacol Sci.

